# Supplementary material for: MetaRibo-Seq measures translation in microbiomes
Source: Nat Commun. 2020 Jun 29;11:3268. doi: 10.1038/s41467-020-17081-z (PMC7324362; doi:10.1038/s41467-020-17081-z)
Supplement: Supplementary file 10 — Supplementary Data 7 [file 41467_2020_17081_MOESM10_ESM.zip › File2/Confidence_High_Taxonomy/275463_out.krona.html]

Javascript must be enabled to view this page.

members
magnitude
magnitudeUnassigned
count
unassigned
taxon
rank

275463\_out

342

superkingdom
2
342

976
340
phylum

200643
340
class

340
171549
order

1853231
1
family

genus
283168
1

species
28118

SRS021219\_contig\_number\_9739
1

family
339
815

genus
7

SRS018836\_contig\_number\_contig-100\_861.163942SRS019030\_contig\_number\_contig-100\_1064.115206SRS022524\_contig\_number\_contig-100\_1272.87291SRS050422\_contig\_number\_contig-100\_661.192739SRS053356\_contig\_number\_47976SRS056273\_contig\_number\_32394SRS893292\_contig\_number\_contig-100\_1940.58144
816
339

species
671267

SRS013687\_contig\_number\_31899
1

species
1263040
1

SRS1041143\_contig\_number\_1412

310298
1

SRS014948\_contig\_number\_23578
species

species
1262750

SRS055966\_contig\_number\_1059
1


SRS046068\_contig\_number\_966
1
820
species

species
4

SRS052027\_contig\_number\_3509SRS063370\_contig\_number\_2533SRS063813\_contig\_number\_3584SRS098881\_contig\_number\_7097
818


SRS145308\_contig\_number\_2075SRS893253\_contig\_number\_453SRS893378\_contig\_number\_21436
3
28116
species

species
2292941
1

SRS078176\_contig\_number\_12381


SRS104975\_contig\_number\_15371
1
246787
species

species
29523

SRS104485\_contig\_number\_164SRS143991\_contig\_number\_21334SRS149244\_contig\_number\_12076
3

species

SRS016954\_contig\_number\_126SRS023914\_contig\_number\_14795SRS024075\_contig\_number\_5176SRS076876\_contig\_number\_103SRS077552\_contig\_number\_11803SRS893358\_contig\_number\_10065SRS893380\_contig\_number\_815
7
46506

2292944
4

SRS1055038\_contig\_number\_1712SRS143895\_contig\_number\_38071SRS893252\_contig\_number\_18851SRS971275\_contig\_number\_10114
species

species
817

SRS047044\_contig\_number\_18292
1

species
1263038

SRS1041141\_contig\_number\_1990
1

357276
1

SRS021153\_contig\_number\_673
species


SRS078665\_contig\_number\_2839
1
310297
species

species

SRS142781\_contig\_number\_754
1
371601


SRS050998\_contig\_number\_16991SRS103987\_contig\_number\_3956SRS1041118\_contig\_number\_6611
3
47678
species

species
162156

SRS020622\_contig\_number\_698SRS1041164\_contig\_number\_715
2

species
1

SRS024009\_contig\_number\_6966
28111

204516

SRS016989\_contig\_number\_13268
1
species

species
18

SRS014613\_contig\_number\_431SRS015794\_contig\_number\_contig-100\_1251.91085SRS020394\_contig\_number\_13033SRS045713\_contig\_number\_209SRS048060\_contig\_number\_10837SRS064232\_contig\_number\_2457SRS064276\_contig\_number\_253SRS075963\_contig\_number\_contig-100\_717.717SRS077127\_contig\_number\_17426SRS101433\_contig\_number\_contig-100\_1376.111001SRS1041038\_contig\_number\_14SRS1041136\_contig\_number\_22653SRS1041145\_contig\_number\_38650SRS1055043\_contig\_number\_2932SRS1055067\_contig\_number\_11365SRS1055099\_contig\_number\_6016SRS147653\_contig\_number\_8080SRS893342\_contig\_number\_contig-100\_1975.64083
2292935

species
821

SRS011084\_contig\_number\_11247SRS011134\_contig\_number\_42239SRS011239\_contig\_number\_5826SRS011405\_contig\_number\_14633SRS012902\_contig\_number\_1197SRS012969\_contig\_number\_16464SRS013158\_contig\_number\_contig-100\_24.42530SRS013382\_contig\_number\_1764SRS013521\_contig\_number\_650SRS013638\_contig\_number\_8049SRS013965\_contig\_number\_29147SRS014287\_contig\_number\_20185SRS014313\_contig\_number\_221SRS014412\_contig\_number\_4822SRS014459\_contig\_number\_24775SRS014534\_contig\_number\_503SRS014683\_contig\_number\_6308SRS014736\_contig\_number\_7283SRS014855\_contig\_number\_5177SRS014979\_contig\_number\_33022SRS015065\_contig\_number\_37146SRS015190\_contig\_number\_1755SRS015217\_contig\_number\_16671SRS015369\_contig\_number\_8524SRS015431\_contig\_number\_40849SRS015486\_contig\_number\_311SRS015578\_contig\_number\_18174SRS015663\_contig\_number\_2800SRS015694\_contig\_number\_23229SRS015782\_contig\_number\_44924SRS015854\_contig\_number\_20419SRS015960\_contig\_number\_22390SRS016056\_contig\_number\_2221SRS016095\_contig\_number\_22946SRS016132\_contig\_number\_contig-100\_1068.29278SRS016203\_contig\_number\_11458SRS016335\_contig\_number\_29632SRS016495\_contig\_number\_18993SRS016753\_contig\_number\_10013SRS016840\_contig\_number\_1573SRS016897\_contig\_number\_104SRS017247\_contig\_number\_661SRS017307\_contig\_number\_30666SRS017375\_contig\_number\_287SRS017433\_contig\_number\_10265SRS017521\_contig\_number\_15439SRS017745\_contig\_number\_1056SRS017859\_contig\_number\_1050SRS018351\_contig\_number\_12803SRS018427\_contig\_number\_31478SRS018623\_contig\_number\_3393SRS018817\_contig\_number\_13308SRS018888\_contig\_number\_6103SRS018936\_contig\_number\_12609SRS019068\_contig\_number\_37422SRS019161\_contig\_number\_23990SRS019178\_contig\_number\_1683SRS019267\_contig\_number\_568SRS019381\_contig\_number\_13122SRS019397\_contig\_number\_1942SRS019445\_contig\_number\_30556SRS019496\_contig\_number\_2329SRS019601\_contig\_number\_25436SRS019693\_contig\_number\_1355SRS019808\_contig\_number\_20360SRS019910\_contig\_number\_25136SRS019968\_contig\_number\_6657SRS020328\_contig\_number\_7001SRS020394\_contig\_number\_contig-100\_2300.96230SRS020508\_contig\_number\_300SRS021484\_contig\_number\_27969SRS021948\_contig\_number\_1089SRS022713\_contig\_number\_658SRS023526\_contig\_number\_12872SRS023829\_contig\_number\_194SRS023971\_contig\_number\_753SRS024492\_contig\_number\_16250SRS024625\_contig\_number\_18296SRS042284\_contig\_number\_2993SRS042966\_contig\_number\_243SRS043411\_contig\_number\_515SRS043667\_contig\_number\_6530SRS044535\_contig\_number\_18936SRS045004\_contig\_number\_463SRS045244\_contig\_number\_1953SRS045645\_contig\_number\_15290SRS045739\_contig\_number\_966SRS046369\_contig\_number\_12288SRS046502\_contig\_number\_10320SRS046810\_contig\_number\_contig-100\_46.47SRS047014\_contig\_number\_13188SRS047433\_contig\_number\_21653SRS048164\_contig\_number\_14835SRS049402\_contig\_number\_14284SRS049446\_contig\_number\_21SRS049712\_contig\_number\_24575SRS049773\_contig\_number\_105SRS049900\_contig\_number\_10987SRS049959\_contig\_number\_52504SRS049995\_contig\_number\_7815SRS050026\_contig\_number\_8495SRS050520\_contig\_number\_65SRS050752\_contig\_number\_30660SRS050801\_contig\_number\_contig-100\_159.28206SRS050941\_contig\_number\_9762SRS050974\_contig\_number\_1540SRS051031\_contig\_number\_contig-100\_108.194967SRS051610\_contig\_number\_2838SRS051882\_contig\_number\_16842SRS052576\_contig\_number\_712SRS052697\_contig\_number\_9981SRS053087\_contig\_number\_contig-100\_98.44544SRS053214\_contig\_number\_4103SRS053335\_contig\_number\_90SRS053398\_contig\_number\_14839SRS054059\_contig\_number\_contig-100\_498.45868SRS054352\_contig\_number\_8768SRS054905\_contig\_number\_20752SRS055017\_contig\_number\_37562SRS056537\_contig\_number\_18470SRS057478\_contig\_number\_5564SRS057480\_contig\_number\_3362SRS058268\_contig\_number\_contig-100\_1.2SRS058723\_contig\_number\_6071SRS062427\_contig\_number\_7379SRS062654\_contig\_number\_14495SRS062701\_contig\_number\_6146SRS063127\_contig\_number\_846SRS063190\_contig\_number\_13429SRS063489\_contig\_number\_4158SRS063518\_contig\_number\_15740SRS063928\_contig\_number\_326SRS064557\_contig\_number\_9821SRS064645\_contig\_number\_1670SRS064973\_contig\_number\_201SRS065176\_contig\_number\_1330SRS065397\_contig\_number\_8954SRS074586\_contig\_number\_4572SRS074670\_contig\_number\_1434SRS074964\_contig\_number\_1901SRS075078\_contig\_number\_13136SRS075398\_contig\_number\_1951SRS075716\_contig\_number\_572SRS075773\_contig\_number\_25730SRS075878\_contig\_number\_21566SRS075984\_contig\_number\_3619SRS076756\_contig\_number\_10729SRS076976\_contig\_number\_2835SRS077194\_contig\_number\_21760SRS077231\_contig\_number\_16097SRS077294\_contig\_number\_12098SRS077335\_contig\_number\_7649SRS077392\_contig\_number\_contig-100\_1098.252545SRS077454\_contig\_number\_1706SRS077502\_contig\_number\_3061SRS077589\_contig\_number\_1397SRS077641\_contig\_number\_22065SRS077730\_contig\_number\_6582SRS077849\_contig\_number\_11475SRS077991\_contig\_number\_258SRS078242\_contig\_number\_1298SRS097920\_contig\_number\_923SRS097958\_contig\_number\_18189SRS098061\_contig\_number\_523SRS098073\_contig\_number\_14265SRS098571\_contig\_number\_45038SRS098644\_contig\_number\_14512SRS098827\_contig\_number\_340SRS100021\_contig\_number\_390SRS100907\_contig\_number\_8669SRS101376\_contig\_number\_18152SRS104036\_contig\_number\_3168SRS104084\_contig\_number\_1742SRS1041031\_contig\_number\_19046SRS1041036\_contig\_number\_3841SRS1041037\_contig\_number\_17102SRS1041090\_contig\_number\_1256SRS1041091\_contig\_number\_17994SRS1041092\_contig\_number\_contig-100\_26.96894SRS1041095\_contig\_number\_2471SRS1041116\_contig\_number\_7396SRS1041129\_contig\_number\_11066SRS1041130\_contig\_number\_1406SRS1041133\_contig\_number\_28770SRS1041134\_contig\_number\_11427SRS1041137\_contig\_number\_contig-100\_1132.176553SRS1041138\_contig\_number\_4064SRS1041139\_contig\_number\_2763SRS1041140\_contig\_number\_19501SRS1041142\_contig\_number\_1439SRS1041144\_contig\_number\_2108SRS1041147\_contig\_number\_4558SRS1041157\_contig\_number\_5202SRS104327\_contig\_number\_6203SRS104400\_contig\_number\_54793SRS104693\_contig\_number\_2139SRS104912\_contig\_number\_16643SRS105082\_contig\_number\_11583SRS1054716\_contig\_number\_16420SRS1054928\_contig\_number\_contig-100\_332.42168SRS1055022\_contig\_number\_5666SRS1055049\_contig\_number\_11901SRS1055056\_contig\_number\_12983SRS1055069\_contig\_number\_469SRS1055076\_contig\_number\_9813SRS1055083\_contig\_number\_6301SRS1055095\_contig\_number\_945SRS140492\_contig\_number\_202SRS142503\_contig\_number\_21453SRS142542\_contig\_number\_1111SRS142599\_contig\_number\_4798SRS142890\_contig\_number\_29103SRS142923\_contig\_number\_23SRS142980\_contig\_number\_6099SRS143070\_contig\_number\_4056SRS143085\_contig\_number\_778SRS143148\_contig\_number\_25979SRS143342\_contig\_number\_413SRS143372\_contig\_number\_5493SRS143417\_contig\_number\_494SRS143523\_contig\_number\_23233SRS143722\_contig\_number\_13332SRS143780\_contig\_number\_4785SRS143876\_contig\_number\_10115SRS144183\_contig\_number\_41754SRS144297\_contig\_number\_8548SRS144362\_contig\_number\_9292SRS144506\_contig\_number\_41989SRS144537\_contig\_number\_33980SRS144603\_contig\_number\_11482SRS144714\_contig\_number\_28212SRS144753\_contig\_number\_8557SRS145497\_contig\_number\_15983SRS146764\_contig\_number\_2478SRS146813\_contig\_number\_4051SRS147022\_contig\_number\_927SRS147039\_contig\_number\_3977SRS147271\_contig\_number\_652SRS147346\_contig\_number\_29364SRS147377\_contig\_number\_697SRS147425\_contig\_number\_477SRS147614\_contig\_number\_7842SRS147766\_contig\_number\_1920SRS147977\_contig\_number\_764SRS148091\_contig\_number\_14181SRS148159\_contig\_number\_55087SRS148196\_contig\_number\_41499SRS148253\_contig\_number\_2507SRS148424\_contig\_number\_27758SRS148784\_contig\_number\_23696SRS148817\_contig\_number\_8238SRS148970\_contig\_number\_651SRS149075\_contig\_number\_12884SRS149181\_contig\_number\_13326SRS149325\_contig\_number\_12532SRS892882\_contig\_number\_16826SRS893170\_contig\_number\_7853SRS893172\_contig\_number\_5747SRS893187\_contig\_number\_15827SRS893256\_contig\_number\_567SRS893259\_contig\_number\_3307SRS893270\_contig\_number\_25137SRS893279\_contig\_number\_16640SRS893285\_contig\_number\_453SRS893288\_contig\_number\_569SRS893300\_contig\_number\_11933SRS893327\_contig\_number\_4737SRS893341\_contig\_number\_14837SRS893342\_contig\_number\_9589SRS893366\_contig\_number\_26318SRS893369\_contig\_number\_8236SRS893373\_contig\_number\_27096SRS893382\_contig\_number\_2032SRS893383\_contig\_number\_10164
274

1239
1
phylum

186801
1
class

186802
1
order

family
1
541000

genus
1263
1

species

SRS016132\_contig\_number\_1313
1
40518

phylum
74201
1

class
203494
1

order
1
48461

1
1647988
family

genus
1
239934

239935

SRS075821\_contig\_number\_97
1
species
